# Microbiological, clinical and molecular findings of non-typhoidal *Salmonella* bloodstream infections associated with malaria, Oriental Province, Democratic Republic of the Congo

**DOI:** 10.1186/s12879-016-1604-1

**Published:** 2016-06-10

**Authors:** Dadi Falay, Laura Maria Francisca Kuijpers, Marie-France Phoba, Hilde De Boeck, Octavie Lunguya, Emmanuel Vakaniaki, Sophie Bertrand, Wesley Mattheus, Pieter-Jan Ceyssens, Raymond Vanhoof, Hugo Devlieger, Chris Van Geet, Erik Verheyen, Dauly Ngbonda, Jan Jacobs

**Affiliations:** Department of Pediatrics, University Hospital of Kisangani, Kisangani, the Democratic Republic of the Congo; Department of Clinical Sciences, Institute of Tropical Medicine, Antwerp, Belgium; Department of Microbiology and Immunology, KU Leuven, Leuven, Belgium; Department of Clinical Microbiology, National Institute for Biomedical Research, Kinshasa, the Democratic Republic of the Congo; General Referral Hospital of Kabondo, Kisangani, the Democratic Republic of the Congo; Belgian National Centre for Salmonella, Scientific Institute of Public Health, Brussels, Belgium; Department of Pediatrics, University Hospital of Leuven, KU Leuven, Leuven, Belgium; OD Taxonomy & Phylogeny, Royal Belgian Institute of Natural Sciences, Brussels, Belgium; Evolutionary Ecology, University of Antwerp, Antwerp, Belgium

**Keywords:** Bloodstream infections, Salmonella, Democratic Republic of the Congo, Antibiotic, Molecular typing, Symptoms, Children, Malaria

## Abstract

**Background:**

In sub-Saharan Africa, non-typhoidal *Salmonella* (NTS) can cause bloodstream infections, referred to as invasive non-typhoidal *Salmonella* disease (iNTS disease); it can occur in outbreaks and is often preceded by malaria. Data from Central Africa is limited.

**Methods:**

Clinical, microbiological and molecular findings of NTS recovered in a blood culture surveillance project (2009–2014) were analyzed.

**Results:**

In March-July 2012 there was an epidemic increase in malaria infections in the Oriental Province of the Democratic Republic of the Congo (DRC). In one referral hospital, overall hospital admissions in June 2012 were 2.6 times higher as compared to the same period in the years before and after (336 versus an average of 128 respectively); numbers of malaria cases and blood transfusions were nearly three- and five-fold higher respectively (317 versus 112 and 250 versus 55). Case fatality rates (in-hospital deaths versus all admissions) peaked at 14.6 %. *Salmonella* Typhimurium and *Salmonella* Enteritidis together accounted for 88.9 % of pathogens isolated from blood cultures collected during an outreach visit to the affected districts in June 2012. Children infected with *Salmonella* Enteritidis (33 patient files available) tended to be co-infected with *Plasmodium falciparum* more often than children infected with *Salmonella* Typhimurium (40 patients files available) (81.8 % versus 62.5 %). Through the microbiological surveillance project (May 2009–May 2014) 113 unique NTS isolates were collected (28.5 % (113/396) of pathogens); most (95.3 %) were recovered from children < 15 years. *Salmonella* Typhimurium (*n* = 54) and *Salmonella* Enteritidis (*n* = 56) accounted for 47.8 % and of 49.6 % NTS isolates respectively. Multilocus variable-number tandem-repeat analysis (MLVA) revealed more heterogeneity for *Salmonella* Typhimurium than for *Salmonella* Enteritidis. Most (82/96, 85.4 %) NTS isolates that were available for antibiotic susceptibility testing were multidrug resistant. All isolates were susceptible to ceftriaxone and azithromycin.

**Conclusion:**

During the peak of an epidemic increase in malaria in the DRC in 2012, a high proportion of multidrug resistant *Salmonella* Typhimurium and *Salmonella* Enteritidis were isolated from blood cultures. Overall, the two serovars showed subtle differences in clinical presentation and genetic diversity.

**Electronic supplementary material:**

The online version of this article (doi:10.1186/s12879-016-1604-1) contains supplementary material, which is available to authorized users.

## Background

In industrialized countries, non-typhoidal *Salmonella* (NTS, i.e., *Salmonella* serovars other than *Salmonella enterica* serovar Typhi and *Salmonella enterica* serovar Paratyphi) are associated with self-limiting diarrhea. In sub-Saharan Africa however, they often cause bloodstream infections, which is referred to as invasive non-typhoidal *Salmonella* disease (iNTS disease). They are among the pathogens most frequently isolated from blood cultures [1–4]. In fact, iNTS disease poses an important public health problem with an estimated population-based annual incidence of 175–388 cases/100,000 in children aged 3–5 years of age [2]. Due to the lack of microbiological facilities in many African countries, the true burden of iNTS disease is not well known and the condition remains undiagnosed and hence invisible and neglected.

iNTS disease occurs mainly in children and immune-compromised individuals and a number of conditions make individuals more susceptible including malnutrition and *Plasmodium falciparum* malaria [5–9]. The two NTS serovars most prevalent in sub-Saharan Africa are *Salmonella enterica* serovar Typhimurium (hereafter referred to as *Salmonella* Typhimurium) and *Salmonella enterica* serovar Enteritidis (hereafter referred to as *Salmonella* Enteritidis).

**Fig. 1:**
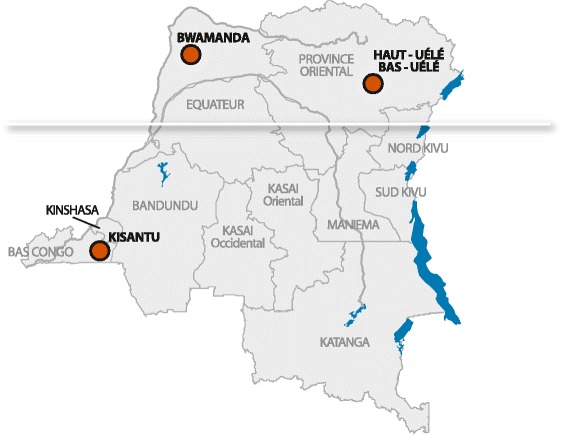
Map of DRC with the three places recently affected by a microbiologically documented (epidemic) increase in *Salmonella *infections (i) Kisantu [15]; (ii) Bwamanda [16]; (iii) districts of Bas-Uélé and Haut-Uélé (present report). © Institute of Tropical Medicine, Antwerp

Studies about NTS infections in children have been reported mainly from East, West and South Africa [1, 10–14]. Recently, epidemic increases of NTS infections were reported from the Democratic Republic of the Congo (DRC) in Central Africa; in Kisantu in 2011 (South West, Bas Congo Province) and in Bwamanda in 2012 (North West, Equatorial Province) [15, 16] (Fig. 1).

**Fig. 2:**
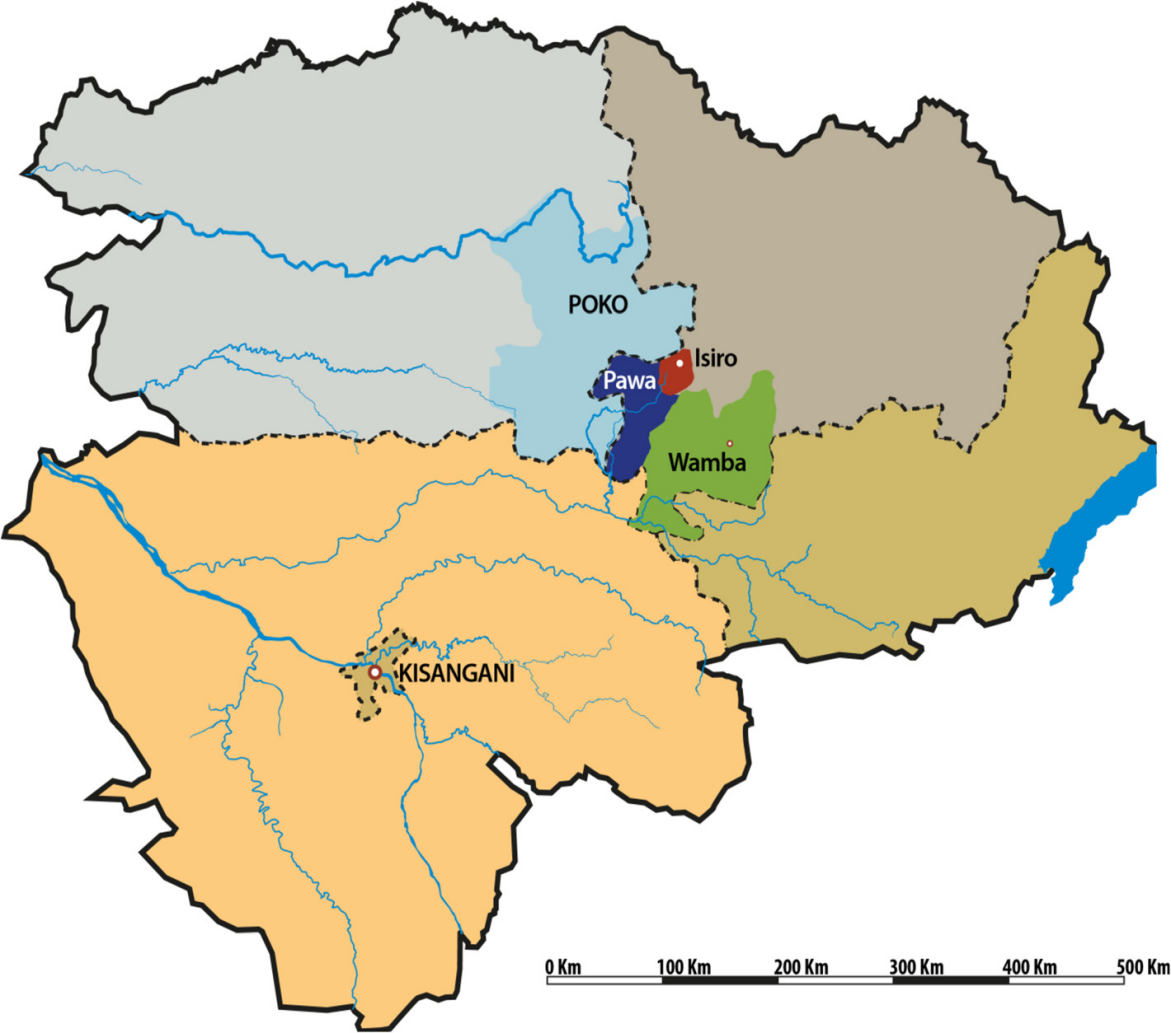
The four health zones affected by the iNTS disease outbreak in 2012: Wamba and Isiro (Haut-Uélé) and Pawa and Poko (Bas-Uélé). © Institute of Tropical Medicine, Antwerp

In April 2012, local health authorities in the district of Haut-Uélé (North East, Oriental Province) (Fig. 2) were alerted by significant increases in numbers of hospitals admissions, blood transfusions and case fatality rates among children. This observation was also made in the neighboring district of Bas-Uélé and was related to an increase of malaria infections as reported by different organizations at that time [17].

However, most children did not respond favorably to antimalarials but improved when antibiotics were administered. This raised suspicion of a concomitant increase in NTS infections as was previously witnessed in Bwamanda and Kisantu and resulted in the Ministry of Health recommending empirical treatment of intravenous quinine combined with antibiotics (ceftriaxone or ciprofloxacin) to all affected children. Blood culture sampling in the affected districts allowed assessment of a possible concomitant burden of invasive *Salmonella* infections. Due to logistic and administrative difficulties, sampling was only initiated from June 2012 onwards. Site visits also included reviewing of clinical charts of pediatric patients with invasive salmonellosis.

The present report aims to describe the clinical, microbiological, and molecular characteristics of the invasive *Salmonella* infections observed during this period of increased admissions and malaria infections in the Bas-Uélé and Haut-Uélé districts as well as of invasive *Salmonella* infections recorded in the entire Oriental Province in the period before and after 2012 (2009–2014).

## Methods

### Study site

The Oriental Province in the DRC covers an area of 503,239 km^2^ with 8,633,573 inhabitants and comprises four administrative districts: Tshopo (including Kisangani, province capital), Haut-Uélé, Bas-Uélé and Ituri. Each district is divided into health zones (HZs) with a General Referral Hospital. The four health zones mainly affected by an epidemic increase in malaria in 2012 were located in the districts Haut-Uélé (HZs of Wamba and Isiro) and Bas-Uélé (HZs of Pawa and Poko) (Fig. 2). Demographic data of these HZs are displayed in Table 1. The majority of the population is living from agriculture, livestock, informal trade and small-scale mining [18]. There is a tropical rainforest climate with peaks of rainfall in April and October and a dryer season between November and February. Malaria is the leading cause of morbidity and mortality among children and *P. falciparum* is holoendemic with a perennial transmission [19].

**Table 1:** Demographic characteristics of the affected health zones (Pawa, Wamba, Poko and Isiro), Oriental Province, DRC

Health zones	HGR capacity (numbers of beds)	Surface (km^2^)	Population (numbers)	HIV prevalence (Pregnant women, %)	Overall acute malnutrition (%)
Pawa	110	2415	157,657	4.7	12.2
Wamba	80	8203	130,473	6.2	10.4
Poko	100	10,876	168,567	5.7	13.3
Isiro	140	1646	208,658	4.5	7.6

*HGR* General Referral Hospital

During the year 2012, the Eastern DRC experienced increases in violence due to various armed groups leading to 466,000 displaced persons in the Oriental Province [20]. Apart from the reported increase in malaria infections, the province also faced outbreaks of cholera, measles and meningitis [21]. The control of these outbreaks was tempered by ongoing insecurity and poor transport infrastructure including the absence of paved roads.

### Blood culture surveillance network, CUKIS, Kisangani

The University Hospital of Kisangani (CUKIS), located in the capital of the Oriental Province, has basic laboratory infrastructure performing blood cultures and is participating in the microbiological surveillance network coordinated by the National Institute of Biomedical Research (INRB) in Kinshasa in collaboration with the Institute of Tropical Medicine (ITM) in Antwerp, Belgium. This network monitors the pathogens involved in bloodstream infections and their antibiotic resistance patterns at various sentinel sites in the DRC. CUKIS receives samples from hospitals and health centers in Kisangani and from more remote sites during outbreak investigations. CUKIS is located at 445 km from the HZ Wamba, 519 km from the HZ Pawa, 571 km from the HZ Isiro and 691 km from the HZ Poko.

Criteria for blood cultures sampling in patients >28 days of age were: (1) a body temperature of ≥38 °C or ≤35.5 °C; (2) suspicion of severe localized infections; or (3) suspicion of sepsis, typhoid fever, and severe malaria. Additional criteria for collection of blood specimens from women in the neonatal period were premature rupture of membranes, intrapartum fever, low Apgar score, or specific symptoms such as tachypnea, cyanosis, and lethargy. For children (≤14 years old), 1–4 mL of blood was sampled into a pediatric blood culture vial (BacT/ALERT PF, bioMérieux, Marcy L’Etoile, France); for adults, 2 × 10 mL of blood was inoculated into aerobic blood culture vials (BacT/ALERT FA, bioMérieux) and incubated at 35 °C for 7 days. Blood cultures were processed according to standard methods as described elsewhere [22, 23]. All isolates were stored in tubes of Trypticase Soya Agar (Oxoid, Basingstoke, UK) and shipped to ITM for confirmation and further identification.

### Serotyping and antibiotic susceptibility

At ITM, isolates biochemically confirmed as *Salmonella* spp. were serotyped using commercial antisera (Sifin, Berlin, Germany). A selection of the isolates were sent to the National Reference Centre in Belgium for confirmation of serotyping by slide agglutination following the Kauffmann-White scheme [24]. Selection of these isolates was based on differences in year and location of collection.

Susceptibility testing of the isolates and interpretation of results were done according to the National Committee for Clinical Laboratory Standards (CLSI) guidelines as described elsewhere [23]. Antibiotic susceptibility tests were performed with the E-test macromethod (bioMérieux) (azithromycin, ciprofloxacin) or disk diffusion (Neo-Sensitabs, Rosco, Taastrup, Denmark) (all other antibiotics). Multidrug resistance (MDR) was defined as co-resistance to all three first-line antibiotics ampicillin, chloramphenicol and trimethoprim-sulfamethoxazole [25].

The methods described apply equally to ongoing surveillance efforts and to the additional site visits done in 2012/2013.

### Determinations of mutations responsible for decreased ciprofloxacin susceptibility

Molecular mechanisms of ciprofloxacin resistance were assessed by amplification and sequencing of the quinolone resistance determining regions (QRDRs) of the *gyrA*, *gyrB*, and *parC* genes as previously described [26]. The fragments were sequenced on an ABI 3130 sequencer. The presence of the plasmid-mediated quinolone resistance *qnr* genes (*qnrA*, *qnrB* and *qnrS*) was determined using PCR [27].

### Multiple-locus variable-number of tandem repeats analysis (MLVA) and assessment of clonality of *Salmonella* Enteritidis and *Salmonella* Typhimurium isolates

To assess the clonal relatedness among *Salmonella* Enteritidis and *Salmonella* Typhimurium isolates, MLVA analysis was performed on all isolates as previously described [28, 29]. For *Salmonella* Enteritidis, profiles were attributed based on the number of tandem repeats on five loci in the following order: SENTR7−, SENTR5−, SENTR6−, SENTR4− and SE3-. For *Salmonella* Typhimurium, profiles were attributed based on the number of tandem repeats on five loci in the following order: STTR9-, STTR5-, STTR6-, STTR10- and STTR3-. Simpson’s index of diversity (D) and Shannon’s indices of diversity (H’) and equitability (E) were calculated to evaluate the discriminatory power and evenness of distribution by using the Biodiversity Calculator [30].

### Clinical data

Outreach visits to the HZs of Pawa, Isiro, Poko and Wamba were performed by an INRB team and a CUKIS collaborator (DF) in June/July 2012 and to the HZ of Pawa in the period October 2012–January 2013. During these and additional visits, clinical data from all children with a documented *Salmonella* bloodstream infection between 2009 and 2014 were retrieved from patient files. Data recorded included age, gender, weight and geographic origin, clinical presentation, diagnosis upon admission, number of blood transfusions, in-hospital outcome, Hb-value (Sahli method [31]) and parasite-based diagnosis of malaria (diagnosed either by microscopy or by rapid diagnostic test). Blood transfusions had been administered according to the criteria established by the national program of blood transfusion [32], i.e., Hb value below 6 g/dl or signs of respiratory and circulatory failure. Anemia was defined as an Hb-value < 11 g/dl and severe anemia was defined as an Hb-value < 5 g/dl. Nutritional status was assessed by the weight-for-age method which for children under 5 years compares the actual weight of the child to the referral charts indicating the ideal weight-for-age and defines low and very low weight-for-age (≤ −2 SD and ≤−3 SD from the reference respectively) [33]. No data on child height, HIV status or malaria density was available. The patient files of children who did not have a confirmed *Salmonella* bloodstream infection were not reviewed.

Data on hospital admissions and case-fatality rates were collected from the hospital registers and concerned all patients admitted to the pediatric ward. For the General Referral Hospital of Poko, blood transfusion files for the year 2011 could not be retrieved.

### Data on rainfall and climate

Data of rainfall in the Oriental Province (2011–2013) were retrieved from the Global Information and Early Warning System from the Food and Agriculture organization of the United Nations [34].

### Data registration, statistical analysis

Demographic, clinical and microbiological data were entered anonymously into an Excel database (Microsoft, Redmond, WA, USA). Only the first isolate for each patient was considered, isolates recovered from a second blood culture drawn within two weeks after the initial one were considered as duplicates, whereas isolates recovered from a repeat blood culture more than two weeks after the initial one were considered as recurrences (either relapse or repeat infections). Statistical analysis was done with Stata 12 (Stata Corp., College Station, TX, USA). P values and (adjusted) odds ratio’s (OR) were calculated by univariate and multivariate logistic regression analysis. When one group contained zero observations for a particular variable, the p value was calculated with a two tailed Fisher’s exact test instead.

It was decided only to keep the categorical variables anemia and severe anemia rather than the continuous variable of hemoglobin level (g/dL), because of the limitations of the measurement (Sahli method) and because it seemed more relevant clinically. When multiple variables differed statistically between groups at a level of P <0.100 and were also clinically relevant, they were further assessed in a multivariable logistic regression analysis. Diagnoses upon admission were not included in the multivariate analysis. To assess the influence of malaria positivity, initial analysis was done stratified for malaria positivity. Stepwise backward regression was used to eliminate variables that this did not retain their statistical significance. Likelihood ratio testing guided the selection of the final model. A p value of < 0.05 was considered significant.

### Ethics statement

The study was conducted according to the principles expressed in the Declaration of Helsinki. Ethical approval for the Microbiological Surveillance Study was granted by the Institutional Review Board of the ITM, the Ethics Committee of Antwerp University, and the Ministry of Health of the DRC. In view of the absence of an Ethical committee in Kisangani at the time of the study, written authorization to review the patient files was obtained from the Medical Inspector of the Oriental Province (Reference number: N^0^ 701/DPS/PERS/872/2012) who represents the highest health authority as well as from the Chief Medical Officer of the Haut-Uélé district (Reference number: 771/23/ISP/HU.O/2012). The retrieved clinical data were only accessible to the CUKIS collaborator (DF), who is a medical doctor, and were subsequently entered anonymously in an Excel database with restricted access.

## Results

### Data of hospital admissions, blood transfusions and malaria cases, 2011–2013

**Fig. 3:**
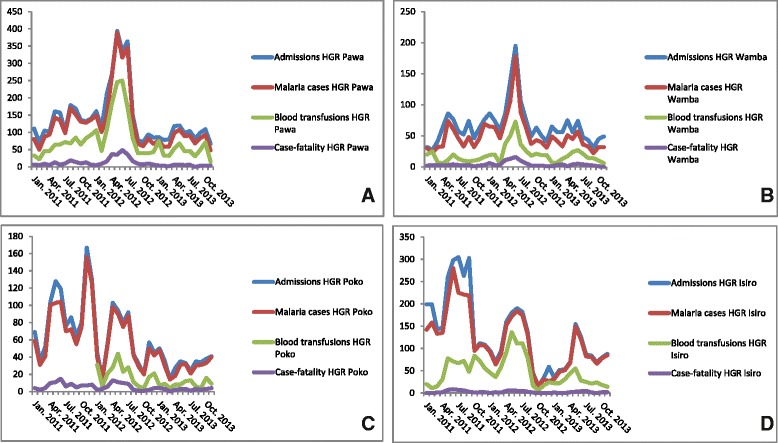
Hospital admissions, malaria cases, blood transfusions and case-fatality rates (2011–2013) for **a** Pawa Health Zone Referral Hospital; **b** Wamba Health Zone Referral Hospital; **c** Poko Health Zone Referral Hospital; **d** Isiro Health Zone Referral Hospital. HGR = General Referal Hospital

**Fig. 4:**
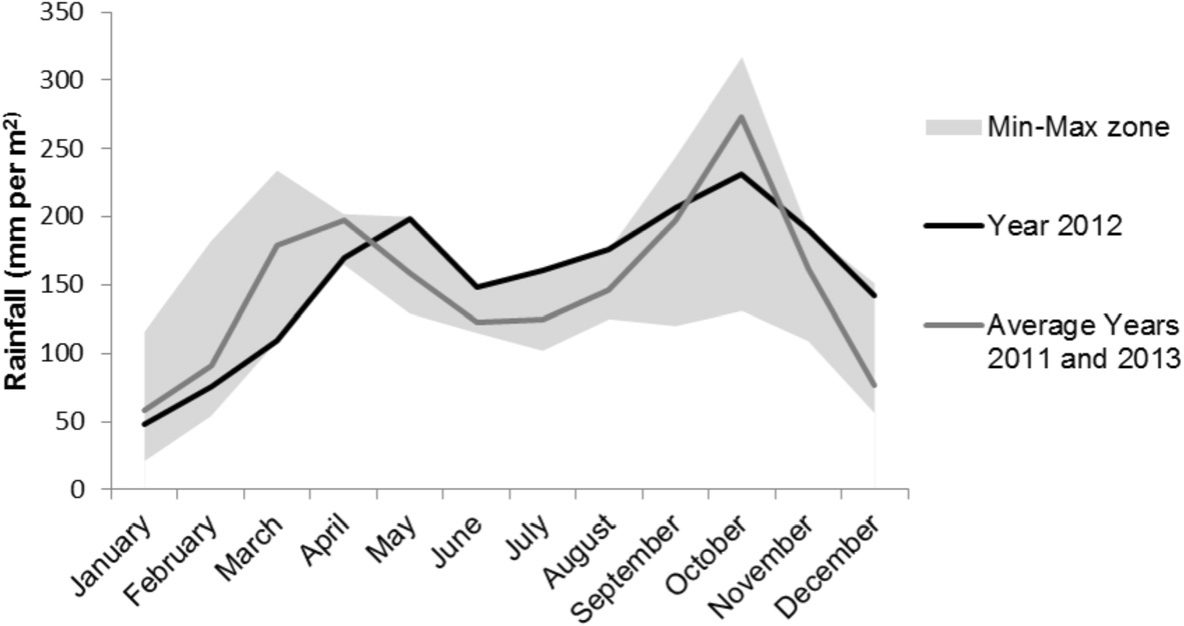
Rainfall in the Oriental Province, DRC (2011–2013). Peak months of the outbreak were April 2012 to June 2012

In Pawa and Wamba HZs, overall hospital admissions increased from March 2012 onwards, reaching a peak in May-June 2012 (Fig. 3a and b). In June 2012, numbers of overall admissions in Pawa were 2.6 times higher as compared to the average of the corresponding month during the years 2011 and 2013 (336 versus 128 respectively). The same was observed in Wamba (195 admissions in June 2012 vs. 77 and 74 in June 2011 and June 2013). The temporal evolution of numbers of malaria cases and blood transfusions aligned with those of the overall hospital admissions: in Pawa, 317 malaria cases were recorded in June 2012, versus an average of 112 for the other years (i.e., a nearly a three-fold increase); for blood transfusions, the difference was almost five-fold (250 versus 55). In Wamba a three-fold increase was seen for the number of malaria cases in June 2012 compared to the average of other years (178 vs. 59), as well as for the number of blood transfusions (73 in June 2012 vs. 24 in other years). The number of case fatalities showed a similar evolution, with a total number of 49 fatalities in Pawa in June 2012 (i.e., an in-hospital case fatality rate of 14.6 % (49/336)) versus an average of 6 cases during the same month in the other years. In Wamba there were 16 fatalities in June 2012 vs. an average of 5 cases that month in other years. The HZs of Poko and Isiro showed a different trend, with a similar peak in June 2012, but with significant peaks in admission numbers and malaria cases already in 2011 (Fig. 3c and d). The increases of 2012 occurred approximately one month after the end of the dryer season, during which less rainfall had been observed compared to other years (Fig. 4).

### NTS infections in the Bas-Uélé and Haut-Uélé districts, 2012-2013

In June 2012, at the request of the Ministry of Health, INRB staff did an outreach visit to all four affected HZs. Out of 87 blood cultures sampled, 18 (20.7 %) grew clinically significant organisms (further referred to as pathogens), of which 16 (88.9 %) were NTS, with *Salmonella* Enteritidis and *Salmonella* Typhimurium accounting for nine and seven isolates respectively. Between October 2012 and January 2013 outreach visit to the HZ of Pawa allowed sampling of another 244 blood cultures, with 38 (15.6 %) pathogens, including *Salmonella* Enteritidis (*n* = 7) and *Salmonella* Typhimurium (*n* = 3) (26.3 % of pathogens). After *Salmonella* species, the most frequently isolated pathogens were *Acinetobacter species* and *Staphylococcus aureus*.

### Blood culture surveillance, CUKIS, Kisangani, *Salmonella* Typhi and NTS isolates (2009 – 2014)

From May 2009 to May 2014, CUKIS processed 3467 blood cultures in total comprising 88.0 % samples (3052/3467) from Kisangani district and 12.0 % (415/3467) from the Haut-Uélé and Bas-Uélé districts, including samples from the outreach visits. In total, 11.4 % (396/3467) blood cultures grew pathogens of which 34.1 % (135/396) were *Salmonella*. The annual proportion varied between 25.0 % (2009–2010) and 42.3 % (2012). *Salmonella* were the most commonly isolated bacteria in both adults and children. Other frequent pathogens were *Staphylococcus aureus* and *Klebsiella pneumoniae.* Among the 135 *Salmonella* isolates, there were five duplicates (all grown within 14 days after the day of first sampling). Of the 130 first (unique) isolates, there were 17 *Salmonella* Typhi and 113 NTS isolates.

### NTS isolates Oriental Province, 2009–2014: serovars and clonality

*Salmonella* Typhimurium and *Salmonella* Enteritidis were the most frequent serovars accounting for approximately half of NTS isolates each (47.8 and 49.6 % out of 113 isolates respectively) (Table 2). In 2011 and 2012, *Salmonella* Enteritidis outnumbered *Salmonella* Typhimurium and vice versa during the years before and after. The other NTS serovars recovered were *Salmonella* Kisangani and *Salmonella* spI 1,4,12:-:1,2 (*Salmonella* group B).

**Table 2:** Distribution of *Salmonella* serovars among clinically significant organisms (CSO), Oriental Province, DRC, 2009–2014. Only first isolates were considered

	2009–2010*	2011	2012	2013	2014**	Total (% of all Salmonella isolates)
	(CSO = 92)	(CSO = 83)	(CSO = 78)	(CSO = 89)	(CSO = 54)	
	n (%)	n (%)	n (%)	n (%)	n (%)	
*Salmonella* Typhi	6	1	2	5	3	17 (13.1)
*Salmonella* Typhimurium	9	6	13	14	12	54 (41.5)
*Salmonella* Enteritidis	6	16	18	13	3	56 (43.1)
Other *Salmonella* serovars***	2	0	0	0	1	3 (2.3)
Total (% of CSO)	23 (25.0)	23 (27.7)	33 (42.3)	32 (35.9)	19 (35.2)	130 (100)

* Data from 2009 and 2010 are grouped together as 2009 was considered as a pilot year (training and implementation)

** For 2014, data were completed till May 27th

*** Including Salmonella group B (one isolate) and Salmonella Kisangani (two isolates)

MLVA analysis of the *Salmonella* Typhimurium isolates revealed heterogeneity with 23 different profiles across 54 isolates collected between 2009 and 2014 (Simpson’s D = 0.95) (Additional file 1: Figure S1). The *Salmonella* Typhimurium isolates also showed an even distribution (Shannon’s H’ = 2.90 and E = 0.93). Forty-four of the 54 *Salmonella* Typhimurium isolates were collected in the Tshopo district (where the provincial capital Kisangani is located), with three major profiles accounting for only 18 isolates (40,9 %) (2-6-11-8-0210 (*n* = 7), 2-5-10-8-0210 (*n* = 6), 2-7-10-8-0210 (*n* = 5)) in this district. Eight *Salmonella* Typhimurium isolates were collected in the Bas-Uélé district. Of these 8, five belonged to the profile 2-8-12-8-0210, which was not isolated in the Tshopo district.

Multilocus variable-number tandem-repeat analysis (MLVA) of the 56 *Salmonella* Enteritidis isolates revealed less heterogeneity with 13 different profiles (Simpson’s D = 0.71) (Additional file 2: Figure S2). Isolates were unevenly distributed over the subtypes, with two major profiles, i.e., 2-13-3-3-NA (*n* = 26) and 2-13-4-3-NA (*n* = 15), accounting for 73.2 % of the tested isolates (Shannon’s H’ = 1.64 and E = 0.64). Thirty-five out of the 56 *Salmonella* Enteritidis isolates were collected in the health district of Tshopo; 26 (74,3 %) of these 35 isolates had the major profile 2-13-3-3-NA. This profile was not isolated in the health districts of Bas-Uélé and Haut-Uélé. Of the 21 *Salmonella* Enteritidis isolates collected in the Bas-Uélé and Haut-Uélé districts between 2009 and 2014, 17 isolates were collected in the year 2012 of which 10 belonged to the second major profile 2-13-4-3-NA.

### Antibiotic resistance of *Salmonella* according to serovar

Susceptibility testing was done for all isolates collected in the DRC between 2009 and 2014 as described elsewhere [6, 23]. Here, we will describe the isolates collected in the Oriental Province during the period 2011–2014 (*n* = 107) as they were not discussed separately from isolates collected in other provinces of the DRC (Table 3; Additional file 3: Table S1).

**Table 3:** Antibiotic resistance rates of 107 invasive *Salmonella* isolates, Oriental Province, DRC, 2011–2014

	*Salmonella* Typhi	*Salmonella* Enteritidis	*Salmonella* Typhimurium	Other NTS^a^
	*n* = 11 (%)	*n* = 50 (%)	*n* = 45 (%)	*n* = 1
Ampicillin	5 (45.5)	44 (88.0)	43 (95.6)	1
TMP-SMX	5 (45.5)	42 (84.0)	41 (91.1)	1
Chloramphenicol	3 (27.3)	43 (86.0)	41 (91.1)	1
MDR	3 (27.3)	41 (82.0)	40 (88.9)	1
DCS	1 (9.1)	0 (0)	1 (2.2)	0
MDR + DCS	0 (0)	0 (0)	1 (2.2)	0
Nalidixic acid	1 (9.1)	0 (0)	1 (2.2)	0

*TMP-SMX* trimethoprim-sulfamethoxazole, *MDR* multi-drug resistant, *DCS* decreased ciprofloxacin susceptibility

^a^ *Salmonella* Group B

In contrast to *Salmonella* Typhi, the majority of NTS (82/96, 85.4 %) were MDR, and with slightly higher resistance rates among *Salmonella* Typhimurium compared to *Salmonella* Enteritidis. Two isolates, one *Salmonella* Typhi and one *Salmonella* Typhimurium (isolated in 2011 and 2013 respectively), showed decreased ciprofloxacin susceptibility. In the *Salmonella* Typhi isolate with a MIC-value of 0.19 μg*/*ml for ciprofloxacin, a *gyrA* resistance mutation (Asp87-Tyr/Glu133-Gly) was detected; the *Salmonella* Typhimurium isolate with a MIC of 0.25 μg*/*ml for ciprofloxacin also showed a *gyrA* resistance mutation (Asp87-Asn). Both isolates were resistant to nalidixic acid.

For all NTS isolates combined, MIC50 and MIC90 values for ciprofloxacin were 0.012 μg/ml and 0.047 μg/ml respectively. The MIC50 value for ciprofloxacin of these isolates was 0.012 μg/ml in the period 2011–2012 and 0.016 μg/ml in the period 2013–2014 while the MIC90 value increased from 0.016 to 0.047 μg*/*ml. All isolates were susceptible to ceftriaxone and azithromycin.

### Clinical data of children with invasive salmonellosis according to serovar (2009–2014)

Clinical charts of children with iNTS diagnosed through ongoing surveillance, as well as during the additional site visits in 2012–2013, were reviewed.

A total of 91/117 (77.8 %) children with invasive salmonellosis were < 5 years old. As expected, the median age for children with iNTS disease (24 months (IQR 12–36)) was significantly lower as compared to children infected with *Salmonella* Typhi (96 months (IQR 48–123); *p* = 0.000).

For 88 out of 124 (71.0 %) children with invasive salmonellosis clinical files were available (Tables 4 and 5). There was a significant difference in diagnosis upon admission between iNTS disease and *Salmonella* Typhi cases; the diagnosis of typhoid fever was raised in 68.8 % of children with confirmed *Salmonella* Typhi infection compared to 18.5 % of children with iNTS disease (*p* = 0.000). This was in line with differences in the clinical presentation: in particular, dyspnea and pallor tended to occur more frequently among children with iNTS disease compared to children with *Salmonella* Typhi infection (46.7 % vs. 23.1 % and 76 % vs. 38.5 % (*p* = 0.010) respectively), while vomiting tended to be less frequent (45.3 % vs. 69.2 %). Bivariate analysis also showed significant differences in malaria positivity (69.3 % vs. 30.8 %; *p* = 0.012), anemia (98.6 % vs. 76.9 %; *p* = 0.010), and rate of blood transfusions (65.3 % vs. 30.8 %; *p* = 0.026) between the children with iNTS disease vs. the children with a *Salmonella* Typhi infection respectively. We included these variables in the multivariate analysis and initially stratified for malaria positivity. In both groups (malaria positives vs. malaria negatives), age (months) was the only variable that remained significant with an OR similar in both groups. We therefore decided to include malaria positivity as a variable in the multivariable analysis. Likelihood ratio testing indicated that the best model included the variables age (adjusted OR 0.95 (95 % CI 0.93–0.97)), malaria positivity (adjusted OR 6.31 (95 % CI 0.99–39.95) and pallor (adjusted OR 5.99 (95 % CI 0.95–37.77). Age remained significant (*p* = 0.000) indicating that younger age is associated with non-typhoidal *Salmonella* infection while malaria positivity and pallor were borderline significant (*p* = 0.050 and *p* = 0.057 respectively).

**Table 4:** Demographic, clinical and laboratory data of children with a *Salmonella* Typhi or non-typhoidal *Salmonella* infection, Oriental Province, DRC, 2009–2014. Except for age, data represent numbers (%)

	*Salmonella Typhi*		Non-typhoidal *Salmonella*		Bivariate analysis		Multivariate analysis	
Characteristics	N	Yes (%)	N	Yes (%)	OR (CI)	p-value	aOR (CI)	p-value
Demographics								
Median age, months (IQR)	16	96 (48–123)	101	24 (12–36)	0.96 (0.94–0.97)	0.000	0.95 (0.93–0.97)	0.000
Male gender	16	7 (43.8)	108	63 (58.3)	1.8 (0.62–5.19)	0.277		
Nutritional data (age <60 months)								
Low weight-for-age	3	0 (0.0)	69	14 (20.3)	*	1.000^b^		
Very low weight-for-age	3	0 (0.0)	69	11 (15.9)	*	1.000^b^		
Symptoms at admission								
Vomiting	13	9 (69.2)	75	34 (45.3)	0.36 (0.10–1.30)	0.121		
Diarrhea	13	2 (15.4)	75	21 (28.0)	2.13 (0.43–10.47)	0.348		
Cough	13	8 (61.5)	75	40 (53.3)	0.71 (0.21–2.38)	0.584		
Dyspnea	13	3 (23.1)	75	35 (46.7)	2.91 (0.74–11.45)	0.125		
Lethargy	13	9 (69.2)	75	44 (58.7)	0.63 (0.17–2.23)	0.475		
Coma	13	0 (0.0)	75	1 (1.3)	*	1.000^b^		
Seizures	13	0 (0.0)	75	7 (9.3)	*	0.588^b^		
Pallor	13	5 (38.5)	75	57 (76.0)	5.06 (1.47–17.44)	0.010	5.99 (0.95–37.77)	0.057
Splenomegaly	13	3 (23.1)	69	18 (26.1)	1.17 (0.29–4.75)	0.820		
Hepatomegaly	13	0 (0.0)	69	13 (18.8)	*	0.115^b^		
Antibiotic exposure <2 weeks	15	5 (33.3)	95	34 (35.8)	1.11 (0.35–3.52)	0.853		
Diagnosis upon admission^c^								
Malaria	16	5 (31.3)	107	58 (54.2)	2.60 (0.84–8.00)	0.088		
Pneumonia	16	1 (6.3)	107	17 (15.7)	2.83 (0.35–22.89)	0.329		
Typhoid fever	16	11 (68.8)	107	20 (18.7)	0.10 (0.03–0.33)	0.000		
Septicemia	16	0 (0.0)	107	13 (12.1)	*	0.214^b^		
Urinary tract infection	16	1 (6.3)	107	7 (6.5)	1.15 (0.12–9.14)	0.965		
Gastro-intestinal tract infection	16	0 (0.0)	107	4 (3.7)	*	1.000^b^		
Meningitis	16	1 (6.3)	107	8 (7.4)	1.2 (0.13–10.28)	0.861		
Other diagnosis	16	5 (31.3)	107	13 (12.0)	0.30 (0.09–1.00)	0.051		
Laboratory data								
Malaria diagnosis^a^	13	4 (30.8)	75	52 (69.3)	5.08 (1.42–18.22)	0.012	6.31 (0.99–39.95)	0.050
Anemia (Hb < 11 g/dl)	13	10 (76.9)	74	73 (98.6)	21.9 (2.07–231.40)	0.010		
Severe anemia (Hb < 5 g/dl)	13	1 (7.7)	74	22 (29.7)	5.07 (0.62–41.45)	0.129		
Transfusion/Outcome								
Blood transfusion	13	4 (30.8)	75	49 (65.3)	4.24 (1.19–15.10)	0.026		
Died in hospital	13	1 (7.7)	75	10 (13.3)	1.84 (0.21–15.78)	0.575		

*OR* Odd’s’ratio, *aOR* adjusted Odd’s ratio

^*^Predicts failure perfectly

^a^Malaria diagnosis as confirmed by either thick blood film microscopy or rapid diagnostic test

^b^Fisher exact test was used to calculate p value (other variables: logistic regression)

^c^Diagnoses upon admission were not included in the multivariate analysis

**Table 5:** Demographic, clinical and laboratory data of children with a *Salmonella* Typhimurium or *Salmonella* Enteritidis infection, Oriental Province, DRC, 2009–2014. Except for age, data represent numbers (%)

	*Salmonella* Typhimurium		*Salmonella* Enteritidis		Bivariate analysis	
Characteristics	N	Yes (%)	N	Yes (%)	OR (CI)	p-value
Demographics						
Median age, months (IQR)	51	18 (12–30)	47	24 (12–48)	1.00 (0.99–1.02)	0.275
Male gender	53	27 (50.9)	52	33 (63.5)	1.66 (0.77–3.56)	0.188
Nutritional data (age <60 months)						
Low weight-for-age	38	8 (21.1)	30	6 (20.0)	0.93 (0.28–3.07)	0.915
Very low weight-for-age	38	6 (15.8)	30	5 (16.7)	1.06 (0.29–3.90)	0.922
Symptoms at admission						
Vomiting	40	17 (42.5)	33	16 (48.5)	1.27 (0.50–3.21)	0.609
Diarrhea	40	9 (22.5)	33	12 (36.4)	1.96 (0.70–5.49)	0.196
Cough	40	25 (62.5)	33	15 (45.5)	0.5 (0.19–1.27)	0.147
Dyspnea	40	19 (47.5)	33	16 (48.5)	1.04 (0.41–2.61)	0.933
Lethargy	40	22 (55.0)	33	21 (63.6)	1.43 (0.55–3.67)	0.456
Coma	40	1 (2.5)	33	0 (0.0)	*	1.000^b^
Seizures	40	7 (17.5)	33	0 (0.0)	*	0.014^b^
Pallor	40	29 (72.5)	33	28 (84.8)	2.12 (0.65–6.89)	0.210
Splenomegaly	37	7 (18.9)	30	11 (36.7)	2.48 (0.81–7.51)	0.108
Hepatomegaly	37	6 (16.2)	30	7 (23.3)	1.57 (0.46–5.30)	0.466
Antibiotic exposure < 2 week	49	15 (30.6)	44	19 (43.2)	1.72 (0.73–4.03)	0.211
Diagnosis upon admission						
Malaria	52	23 (44.2)	52	34 (65.4)	2.38 (1.07–5.25)	0.032
Pneumonia	52	9 (17.3)	52	7 (13.5)	0.74 (0.25–2.17)	0.588
Typhoid fever	52	11 (21.2)	52	8 (15.4)	0.67 (0.24–1.85)	0.448
Septicemia	52	5 (9.6)	52	8 (15.4)	1.70 (0.51–5.62)	0.378
Urinary tract infection	52	1 (1.9)	52	4 (7.7)	4.25 (0.45–39.38)	0.203
Gastro-intestinal tract infection	52	3 (5.7)	52	1 (1.9)	0.32 (0.03–3.18)	0.331
Meningitis	52	4 (7.7)	52	4 (7.7)	1.00 (0.23–4.23)	1.000
Other diagnosis	52	6 (11.5)	52	7 (13.5)	1.19 (0.37–3.82)	0.767
Laboratory data						
Malaria diagnosis^a^	40	25 (62.5)	33	27 (81.8)	2.7 (0.90–8.04)	0.075
Anemia (Hb < 11 g/dl)	39	38 (97.4)	33	33 (100)	*	1.000^b^
Severe anemia (Hb < 5 g/dl)	39	10 (25.6)	33	12 (36.4)	1.65 (0.60–4.54)	0.327
Transfusion/Outcome						
Blood transfusion	40	25 (62.5)	33	24 (70.6)	1.60 (0.58–4.34)	0.356
Died in hospital	40	7 (17.5)	33	3 (9.1)	0.47 (0.11–1.98)	0.306

*OR* Odd’s’ratio

^*^Predicts failure perfectly

^a^Malaria diagnosis as confirmed by either thick blood film microscopy or rapid diagnostic test

^b^Fisher exact test was used to calculate p value (other variables: logistic regression)

Mortality in children with iNTS disease was 13.3 % (10/75) compared to 7.7 % (1/13) of the children with a *Salmonella* Typhi infection; this did not differ significantly. Seventy per cent (7/10) of deaths due to iNTS disease occurred within the first 24 h after admission.

The median age of children with a *Salmonella* Typhimurium bloodstream infection (18 months (IQR 12–30)) was lower compared to those with a *Salmonella* Enteritidis bloodstream infection (24 months (IQR 12–48)) but this difference was not statistically significant (*p* = 0.128). For all iNTS disease cases combined, malaria represented the most frequent diagnosis upon admission (made in nearly half of cases) followed by, in rank of decreasing frequency, typhoid fever, pneumonia and septicemia. Malaria was suspected more often in children with a *Salmonella* Enteritidis infection compared to children with a *Salmonella* Typhimurium infection (65,4 % vs. 44,2 %; *p* = 0.032).

The most common presenting symptom in children with iNTS disease was pallor, observed in 76 % of all children, followed by lethargy, cough, dyspnea, and vomiting. Diarrhea was reported in only 28 % of the children. The spectrum of symptoms did not differ between the different serovars except for seizures which were only observed in the *Salmonella* Typhimurium group (7 cases only; *p* = 0.014).

Laboratory confirmed *P. falciparum* malaria was recorded in nearly 70 % of all children with iNTS disease; this proportion tended to be higher among the *Salmonella* Enteritidis group compared to the *Salmonella* Typhimurium group (62.5 % vs. 81.8 %; *p* = 0.075).

There was no significant difference in mortality between children infected with *Salmonella* Enteritidis and *Salmonella* Typhimurium.

Overall, 35,5 % (39/110) of patients with a *Salmonella* bloodstream infection reported previous antibiotic exposure.

## Discussion

The present study describes the clinical, microbiological and molecular characteristics of invasive *Salmonella* infections in the Oriental Province during the surveillance period 2009–2014, including data from outreach visits to more remote areas in the province during an epidemic increase of malaria in 2012.

During the surveillance period, the proportion of *Salmonella* among pathogens from blood cultures varied between 25.0 and 42.3 %, which is consistent with other studies from sub-Saharan Africa [2, 35–37]. There was a predominance of serovars *Salmonella* Typhimurium and *Salmonella* Enteritidis that were isolated in equal numbers but with proportions varying over time which is also in line with other studies from sub-Saharan Africa [1, 38, 39]. There was a particular high percentage (88.9 %) of *Salmonella* among the pathogens isolated from blood cultures in the health zones affected by the epidemic increase in malaria in June 2012. During subsequent outreach visits between October 2012-January 2013 (period after the epidemic increase in malaria), this proportion subsequently decreased to only 26.3 %. The latter percentage is more in line with the averages at the provincial level, and suggests that the epidemic increase of malaria was associated with a concomitant increase in *Salmonella* bloodstream infections. Possibly, the data represented here are an underestimation of the actual number of patients co-infected with malaria and NTS. Other studies have shown that case fatalities are higher for children who are co-infected with NTS compared to those infected with malaria alone and, in the present study, part of them might have died before reaching the hospital [40].

The clinical presentation of iNTS disease in this study was dominated by pallor, lethargy, gastro-intestinal and respiratory symptoms. A concomitant infection with pneumococci has been forwarded to explain the prevalence of respiratory symptoms during iNTS disease but the dyspnea could also be related to the high prevalence of anemia among children with iNTS disease or to the acidosis due to sepsis in general [2]. There were no statistically significant differences between the characteristics of *Salmonella* Typhimurium and *Salmonella* Enteritidis bloodstream infections except for occurrence of seizures but this was probably affected by a small sample size.

Children with a *Salmonella* Enteritidis bloodstream infection tended to suffer from concomitant malaria more frequently compared to those infected with *Salmonella* Typhimurium. We are not aware of other studies that pointed to a particular association of *Salmonella* Enteritidis with concurrent malaria infection. More extensive and prospective research is needed to evaluate this finding further.

Overall, more than two-thirds of the children with iNTS disease tested positive for *P. falciparum* malaria which was also previously reported from Bwamanda in the DRC [16]. It is known that *P. falciparum* malaria predisposes to iNTS disease [7–9, 41, 42] and several recent studies have revealed probable underlying mechanisms [7, 43–45]. In the case of the Oriental Province, no clear reason for the upsurge in malaria was identified. The fact that during the end of the dry season less than average rainfall was observed might have played a role in the increase of malaria infections. Also, the influx of persons from conflict areas in the Kivu provinces, that have lower exposure to malaria compared to the Oriental Province (annually averaged infection prevalence of malaria in 2–10 year olds (<5 %) compared to >40 % respectively), might have contributed to the increase [46].

Besides malaria, other known risk factors for contracting iNTS disease are HIV, sickle cell anemia and malnutrition. It is estimated that 12 % of the hospitalized children in the DRC have sickle cell anemia [47]. For HIV, rates among pregnant women in the different districts of the Oriental Province varied between 4.5 and 6.2 % (Table 1). In the study cohort described, severe malnutrition was not very common.

As there was also clinical data available for 13 children with a *Salmonella* Typhi infection, differences with iNTS cases could be explored. Vomiting was more prevalent among children infected with *Salmonella* Typhi compared to those with iNTS disease. Mtove et al. (2010) had similar observations in a study from rural Tanzania (71.3 % of *Salmonella* Typhi cases vs. 53.4 % of iNTS disease cases) [11]. In bivariate analysis, significantly less children infected with *Salmonella* Typhi had pallor, anemia, need for blood transfusion and were less frequently tested positive for malaria compared to the children with iNTS. In the study of Mtove et al. (2010) children with *Salmonella* Typhi infections did not show pallor or severe anemia but children with iNTS did in respectively 62.2 and 40.0 % of the cases [11]. Other studies have also described high numbers of patients with iNTS disease receiving blood transfusions [39, 42]. The final regression model included the variables age, malaria positivity and pallor, but only younger age remained significantly associated with non-typhoidal *Salmonella* infection. These results should be interpreted with caution however given the small sample sizes.

Unlike the epidemic increase of iNTS in Kisantu – which was only identified upon retrospect –the Oriental Province health authorities were alerted to the possibility of an increase in iNTS disease in concurrence with the observed increase in malaria cases. As a consequence, empirical antibiotic treatment was installed early on in the epidemic and this may have accounted for the relatively low case-fatality among the microbiologically confirmed iNTS cases in our cohort. This rate is similar to that found during the outbreak in Bwamanda [16] but lower to that found in Kisantu (23.0 %) [15], the latter being more in line with case fatality rates more frequently reported for iNTS [2, 4]. In addition, given the large surface and geographical inaccessibility of the affected districts in the Oriental Province, with a swamp environment and absence of paved roads, many children are expected to have died at home or during their way to the health care center or hospital. The fact that most documented in-hospital deaths occurred within 24 h after admission supports this hypothesis.

Antibiotic susceptibility testing showed high MDR rates among NTS isolates; only two *Salmonella* isolates showed decreased ciprofloxacin susceptibility*.* However, MIC50 and MIC90 values for ciprofloxacin were higher in the period 2013–2014 compared to the period 2011–2012 which is a worrisome trend.

MLVA profiles of *Salmonella* Enteritidis isolates showed that two closely related profiles accounted for 73.2 % of all the tested isolates. The two major profiles only differed with one tandem repeat at one single locus suggesting very close relatedness [28]. MLVA profiles of the *Salmonella* Enteritidis isolates implicated in the epidemic increases in Kisantu and Bwamanda also showed very close relatedness [28]. Whole genome sequencing of these isolates could provide more insight in the clonality of *Salmonella* Enteritidis associated with iNTS disease.

*Salmonella* Typhimurium isolates were more heterogeneous than *Salmonella* Enteritidis isolates with the two most common MLVA profiles accounting for only 24.1 % of all tested isolates. The major profile isolated from the Haut-Uélé district differed by 1–2 tandem repeats at two loci compared to the major profiles isolated in the Tshopo district. Previous reports on epidemic increases of NTS infections in the DRC did not consider the MLVA profiles for *Salmonella* Typhimurium. A previous study did assess the population structure of 180 *Salmonella* Typhimurium isolates collected in the DRC between 2007 and 2011 with CRISPOL typing and found that >96 % belonged to the CT28 group [48]. This group has a strong association with *Salmonella* Typhimurium multilocus sequence type (ST) 313 which is associated with bloodstream infections and high rates of antimicrobial drug resistance. Also in other Sub-Saharan countries, the majority of isolates are found to be ST 313 [49]. Future research will include genomic sequencing of *Salmonella* Typhimurium isolates from different parts of the DRC, including isolates from the Oriental Province, Kisantu and Bwamanda.

The present study had several limitations, mostly owing to its retrospective nature. First, the outreach visits to the HZs were – due to logistic and administrative constraints – organized at a period considerably after the first alerts of increased admissions and malaria cases. Next, there was no clearly defined population size denominator, precluding calculation of population-based incidence rates. In addition, it could not be ascertained how many children died at home or on their way to the hospital, precluding accurate estimates of case-fatality rates. Furthermore, not all isolates, patients’ files and hospital registers were available for analysis. Other difficulties included accessibility of the affected districts requiring for instance transport of samples by motorbikes on deteriorated roads and subsequent shipment by a small airplane carrier (at a distance of 350 km) to CUKIS, the single diagnostic microbiology laboratory in the Oriental Province.

There were also limitations with regard to laboratory data: no distinction could be made between recent (microscopy negative but rapid diagnostic test positive) versus current malaria (microscopy positive), and the quality of malaria diagnosis probably differed per medical center. The limitations of measuring hemoglobin levels with the Sahli method should be kept in mind especially as no quality control for harmonization was performed.

Finally, it was not possible to compare the clinical characteristics of children with iNTS disease to the other children admitted, precluding a control group. Also, sampling of the affected health districts was mainly restricted to the year 2012, not allowing for comparisons with years before and after.

Sampling during the surveillance period was however constant over time and directed by a set of clinical indications, and clear comparisons with other years could be made. We believe this was also the case for the hospital registers providing compiled data about malaria diagnosis and blood transfusions.

Despite the limitations, this study offers valuable data on a neglected disease from an under investigated area collected during a time of great political instability. Previous articles have highlighted the need for studies that combine epidemiological, microbiological and clinical data on iNTS disease [5]. Last, the study allowed for a comparison between the most dominant NTS serovars.

Future studies should estimate reliable population incidence rates. The development of an (antigen based) rapid diagnostic test would be highly welcome as a tool for diagnosis in low resource settings where microbiological facilities are often absent. More research is also needed on differences between the clinical presentation and outcome of both *Salmonella* Typhimurium and *Salmonella* Enteritidis serovars. Whole genome sequencing of NTS isolates from different areas in the DRC, especially those related to local increases in infections, would provide valuable insights. It is obvious that continued blood culture surveillance is imperative to monitor the proportions and distributions of NTS serovars as well as their antibiotic resistance patterns. Last, efforts to control the increase in malaria in this region should be intensified.

## Conclusion

The present observations confirm NTS as a leading cause of bloodstream infection in an area holoendemic for *P. falciparum* malaria. A sudden increase in hospital admissions among children < 5 years old, associated with a laboratory-confirmed diagnosis of *P. falciparum* malaria needs to raise the suspicion of a concomitant increase in NTS infections, particularly if associated with an increase in blood transfusions and case-fatality.

## Abbreviations

(a)OR, (adjusted) Odd’s ratio; CLSI, National Committee for Clinical Laboratory Standards; CSO, clinically significant organisms; CUKIS, University Hospital of Kisangani; DCS, decreased ciprofloxacin susceptibility; DF, Dadi Falay; DRC, Democratic Republic of the Congo; HGR, General Referral Hospital; HZ, health zone; INRB, National Institute of Biomedical Research; iNTS, invasive non-typhoid *salmonella*; IQR, interquartile range; ITM, Institute of Tropical Medicine; MDR, multidrug resistance; MLVA, multilocus variable-number tandem-repeat analysis; NTS, non-typhoidal *Salmonella*; RDT, rapid diagnostic test; TMP-SMX, trimethoprim-sulfamethoxazole

## Additional files

Additional file 1: Figure S1. Minimum-spanning tree analysis of MLVA (multiple-locus variable-number tandem-repeats analysis) data of 54 *Salmonella* Typhimurium isolates, Oriental Province, DRC, 2009–2014. Each circle in this figure displays a unique MLVA type, with different colours indicating the year of collection of isolates. (PDF 308 kb) Additional file 2: Figure S2. Minimum-spanning tree analysis of MLVA (multiple-locus variable-number tandem-repeats analysis) data of 56 *Salmonella* Enteritidis isolates, Oriental Province, DRC, 2009–2014. Each circle in this figure displays a unique MLVA type, with different colours indicating the year of collection of isolates. (PDF 112 kb) Additional file 3: Table S1. Dataset of isolates collected in the Oriental Province, DRC between 2009 and 2014 that were used for this paper. DCS = Decreased Ciprofloxacin Susceptibility; R = Resistance; ZOI: Zone of inhibition. (XLSX 17 kb)
