# Supplementary material for: Microbiological, clinical and molecular findings of non-typhoidal Salmonella bloodstream infections associated with malaria, Oriental Province, Democratic Republic of the Congo
Source: BMC Infect Dis. 2016 Jun 10;16:271. doi: 10.1186/s12879-016-1604-1 (PMC4902913; doi:10.1186/s12879-016-1604-1)

Figure S1. Minimum-spanning tree analysis of MLVA (multiple-locus variable-number tandem-repeats analysis) data of 54 *Salmonella* Typhimurium isolates from the Oriental Province, DRC, 2009-2014. Each circle displays a unique MLVA type, with different colours indicating the year of collection of isolates.

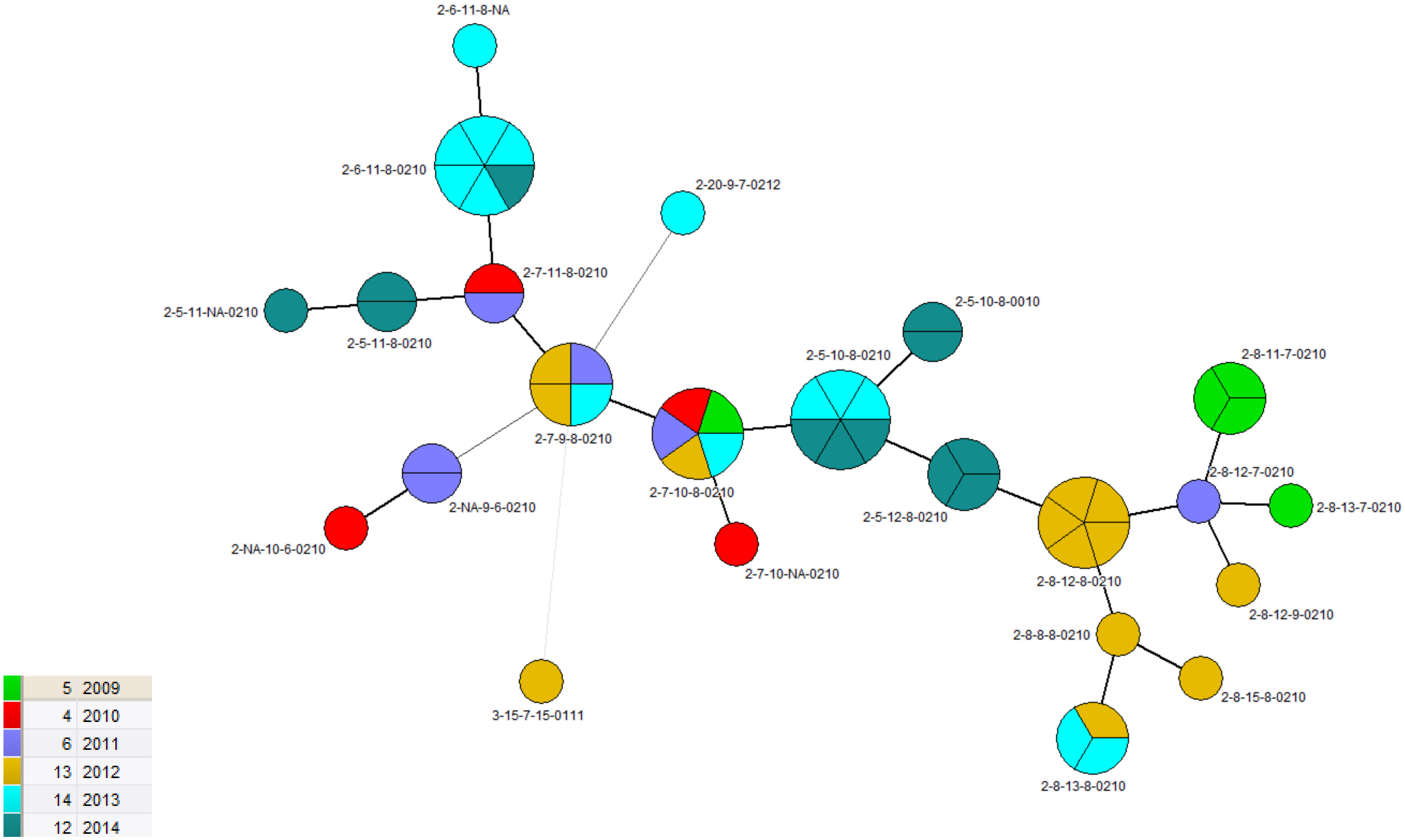

Supplement: Additional file 1: Figure S1. — Minimum-spanning tree analysis of MLVA (multiple-locus variable-number tandem-repeats analysis) data of 54 Salmonella Typhimurium isolates, Oriental Province, DRC, 2009–2014. Each circle in this figure displays a unique MLVA type, with different colours indicating the year of collection of isolates. (PDF 308 kb) [file 12879_2016_1604_MOESM1_ESM.pdf]
