# Supplementary material for: Microbiological, clinical and molecular findings of non-typhoidal Salmonella bloodstream infections associated with malaria, Oriental Province, Democratic Republic of the Congo
Source: BMC Infect Dis. 2016 Jun 10;16:271. doi: 10.1186/s12879-016-1604-1 (PMC4902913; doi:10.1186/s12879-016-1604-1)
Supplement: Additional file 2: Figure S2. — Minimum-spanning tree analysis of MLVA (multiple-locus variable-number tandem-repeats analysis) data of 56 Salmonella Enteritidis isolates, Oriental Province, DRC, 2009–2014. Each circle in this figure displays a unique MLVA type, with different colours indicating the year of collection of isolates. (PDF 112 kb) [file 12879_2016_1604_MOESM2_ESM.pptx]

## Slide 1
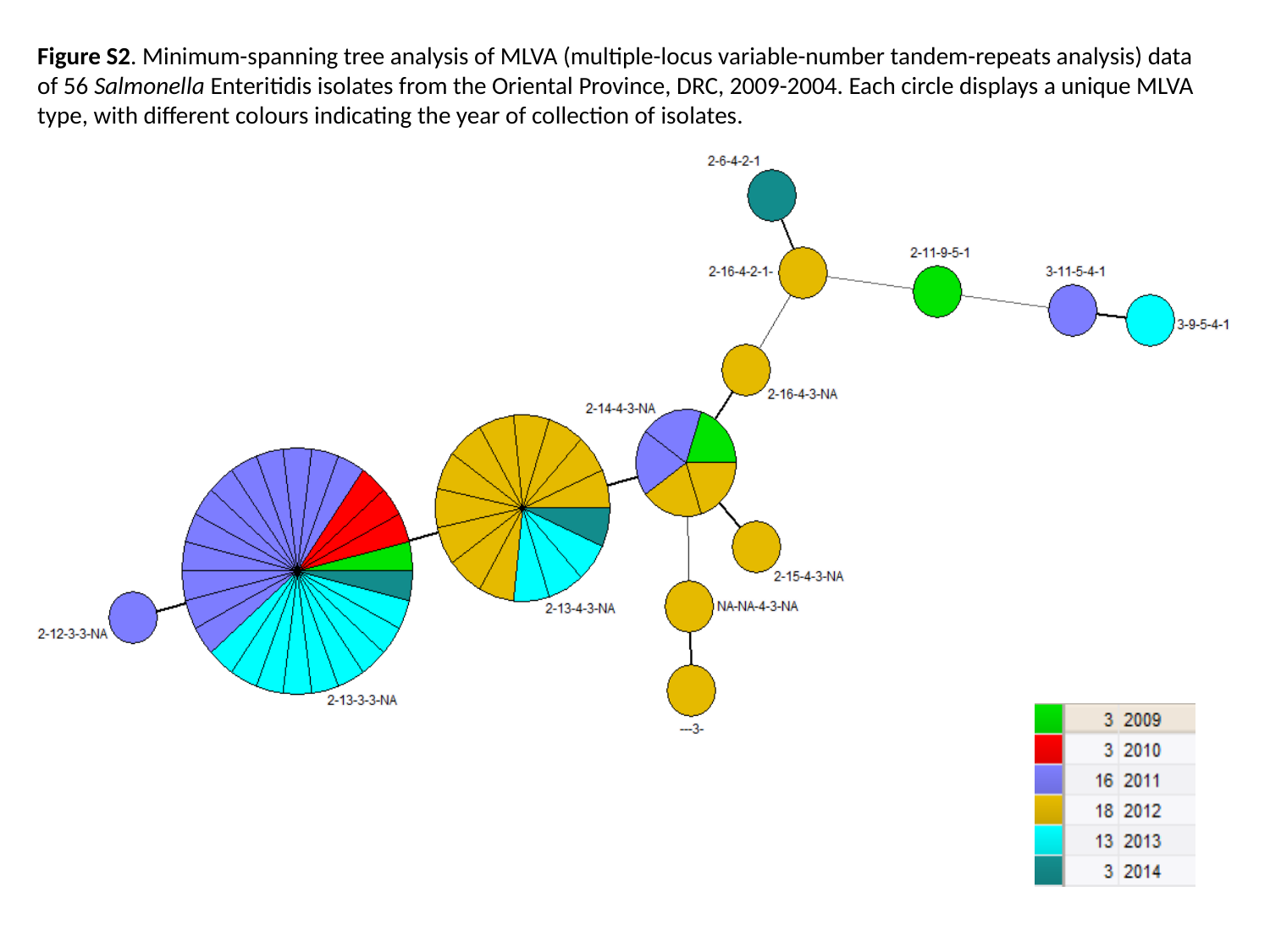

Figure S2. Minimum-spanning tree analysis of MLVA (multiple-locus variable-number tandem-repeats analysis) data of 56 Salmonella Enteritidis isolates from the Oriental Province, DRC, 2009-2004. Each circle displays a unique MLVA type, with different colours indicating the year of collection of isolates.
